# Supplementary material for: Preclinical Therapeutic Assessment of a New Chemotherapeutics [Dichloro(4,4’-Bis(2,2,3,3-Tetrafluoropropoxy) Methyl)-2,2’-Bipryridine) Platinum] in an Orthotopic Patient-Derived Xenograft Model of Triple-Negative Breast Cancers
Source: Pharmaceutics. 2022 Apr 11;14(4):839. doi: 10.3390/pharmaceutics14040839 (PMC9031226; doi:10.3390/pharmaceutics14040839)
Supplement: Supplementary file 1 [file pharmaceutics-14-00839-s001.zip › pharmaceutics-1638448-supplementary-updated.pdf]

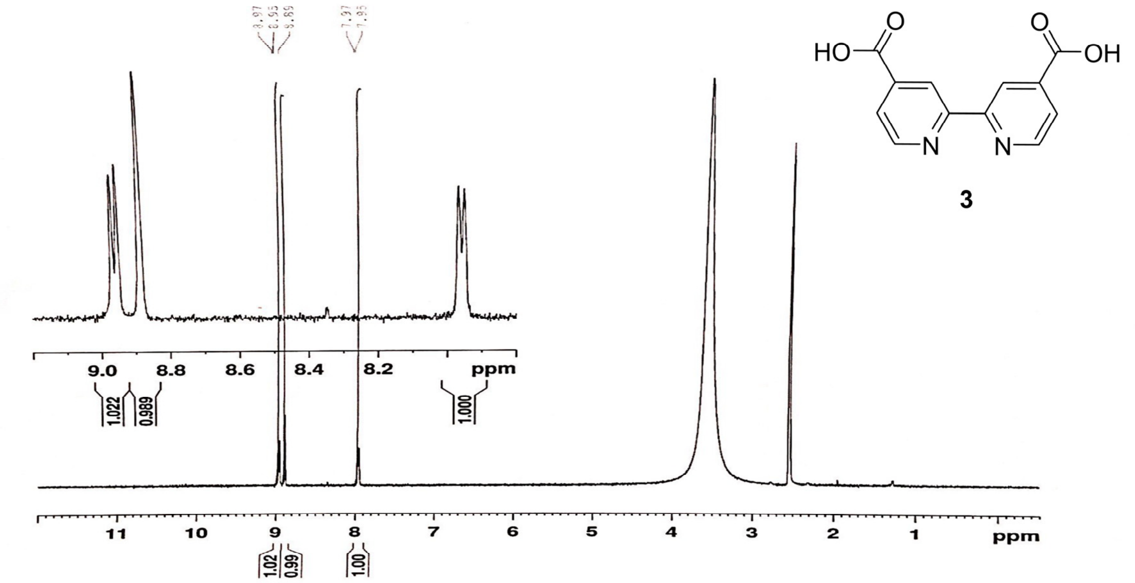

Figure S1. ^{1}H NMR obtained for compound **3** at 300 MHz in DMSO-d_6.

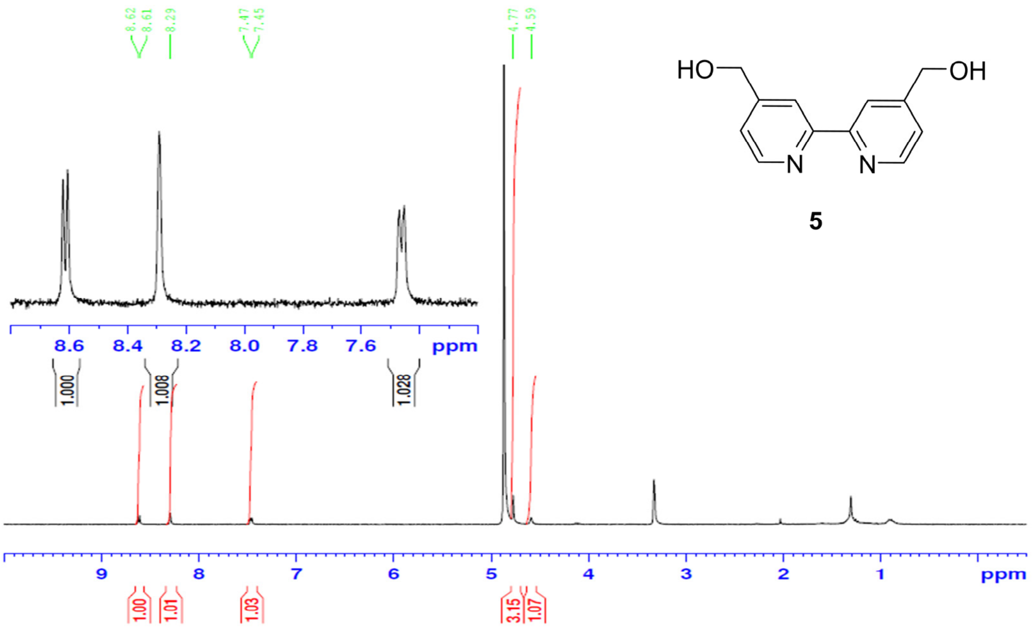

Figure S2. ^{1}H NMR obtained for compound **5** at 300 MHz in CD_3OD.

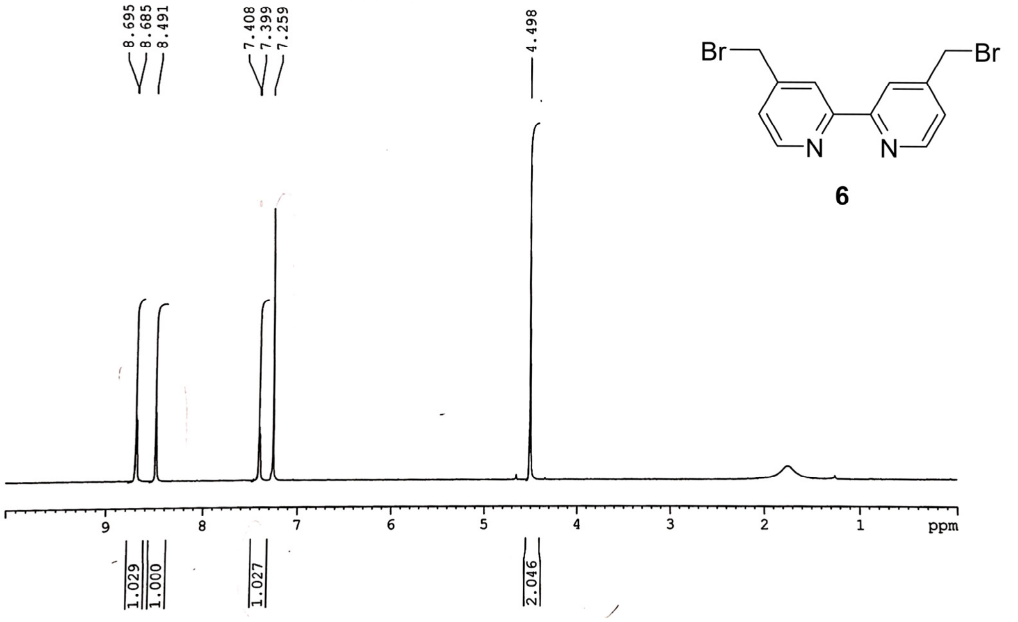

Figure S3. ^1H NMR obtained for compound **6** at 600 MHz in $CDCl_3$.

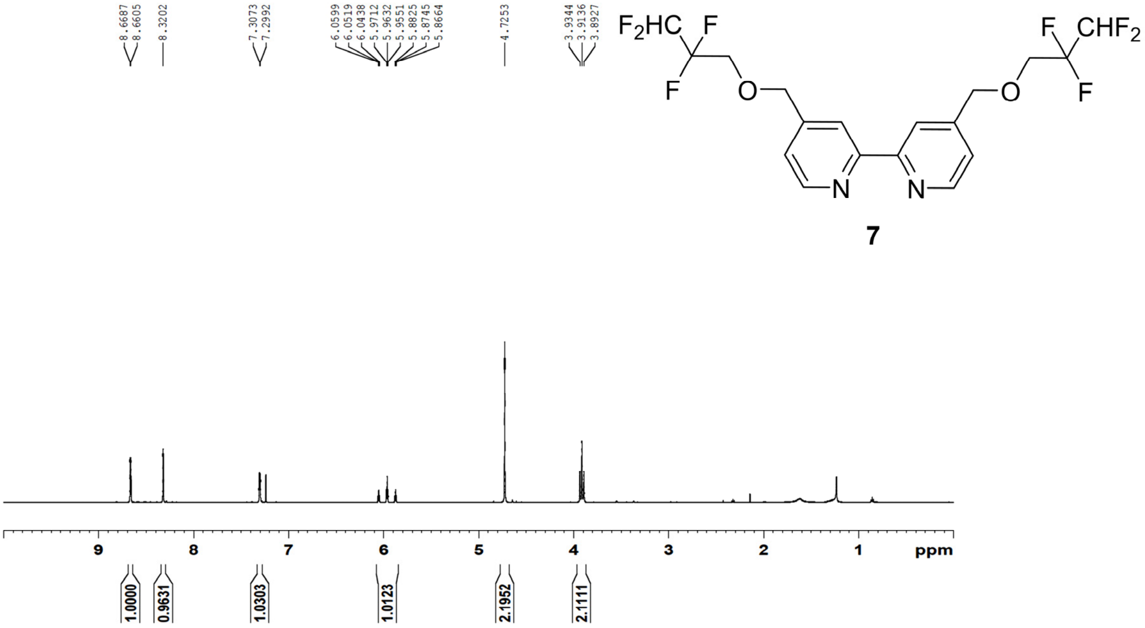

Figure S4. ^1H NMR obtained for compound **7** at 600 MHz in $CDCl_3$.

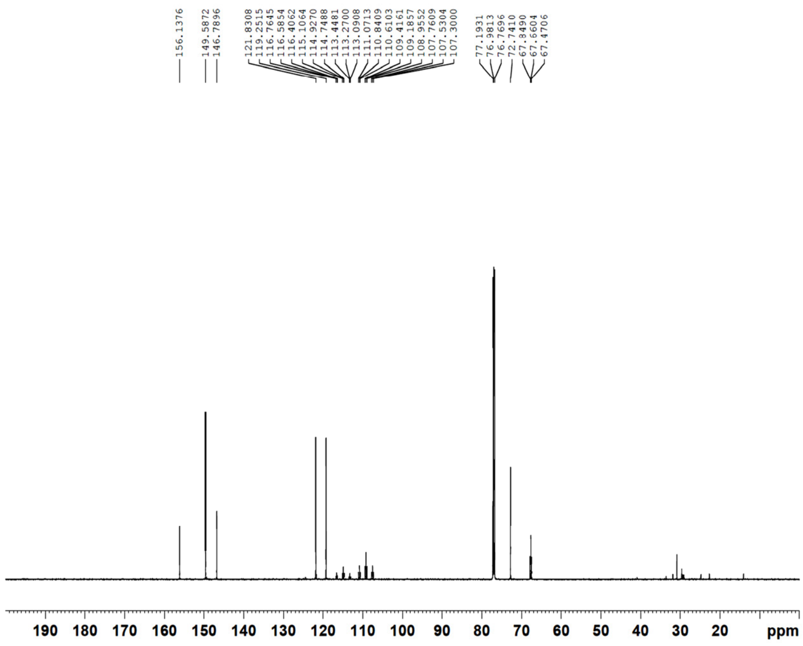

Figure S5. ^{13}C NMR obtained for compound **7** at 150 MHz in $CDCl_3$.

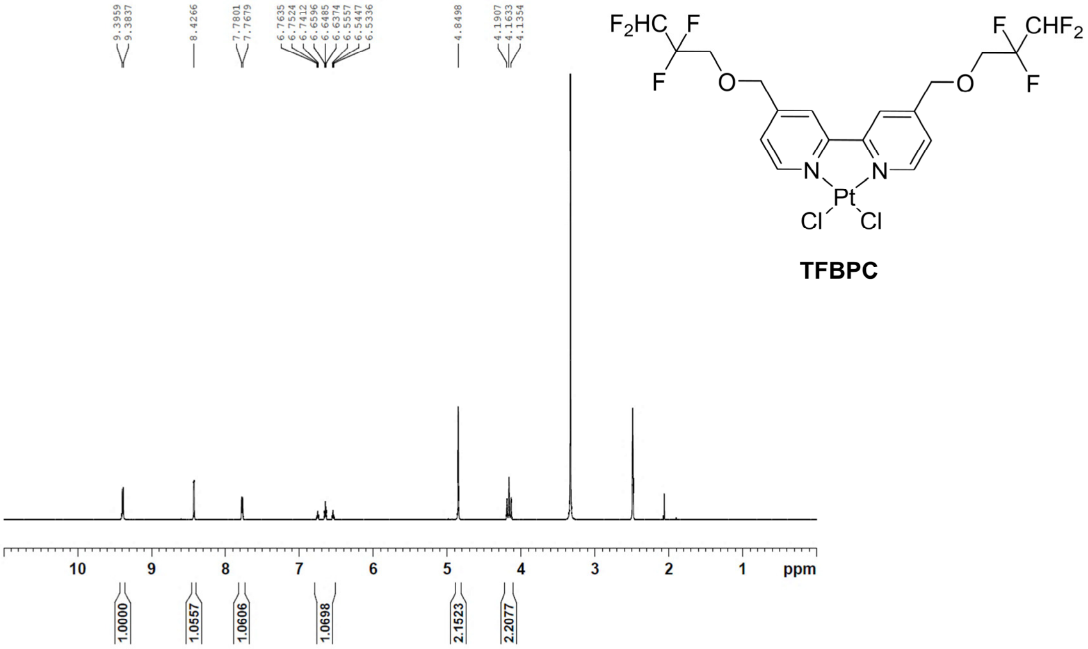

Figure S6. ^{1}H NMR of the compound TFBPC at 500 MHz in DMSO-d_6.

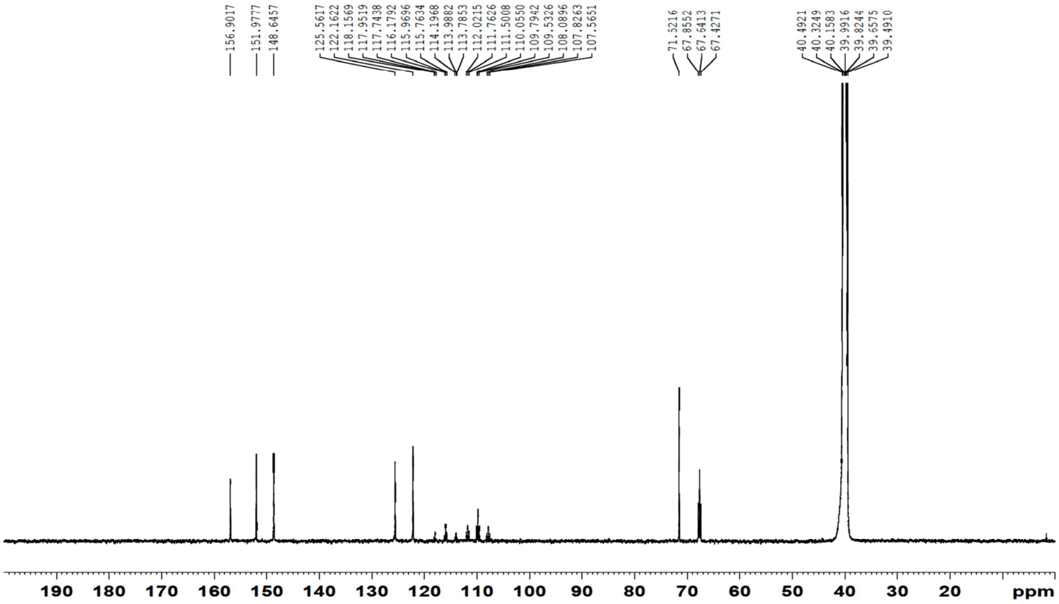

Figure S7. ^{13}C NMR of the compound TFBPC in DMSO-d$_6$.

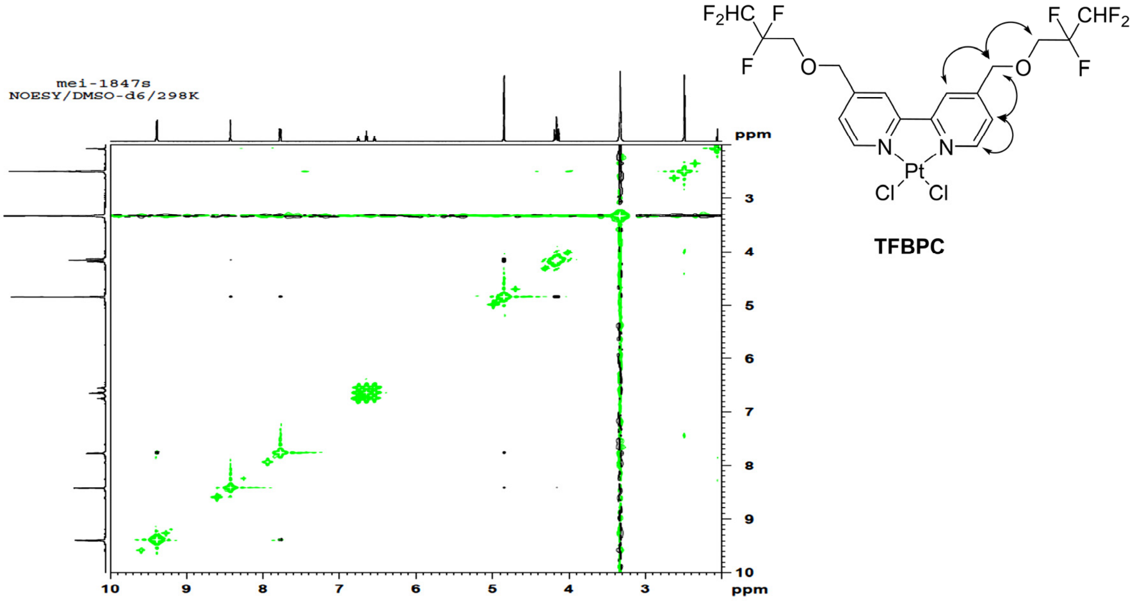

Figure S8. NOESY of compound TFBPC.

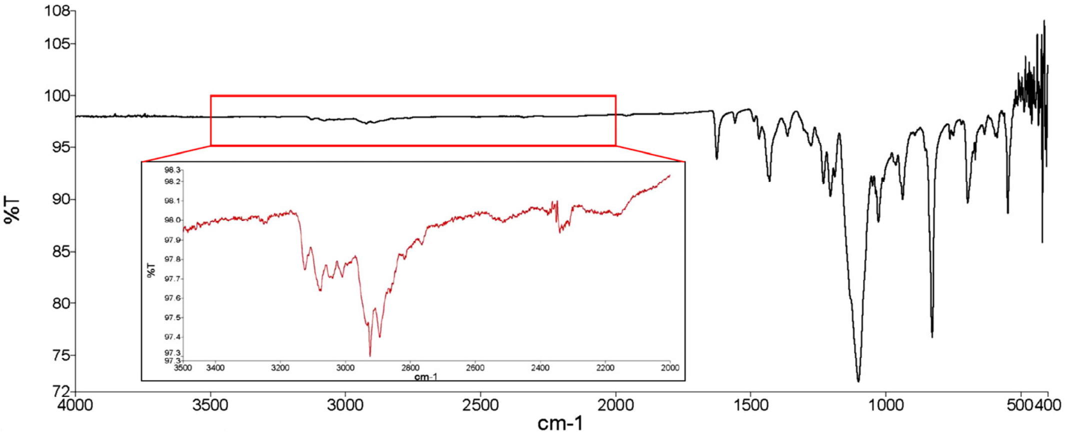

Figure S9. IR spectrum of TFBPC.

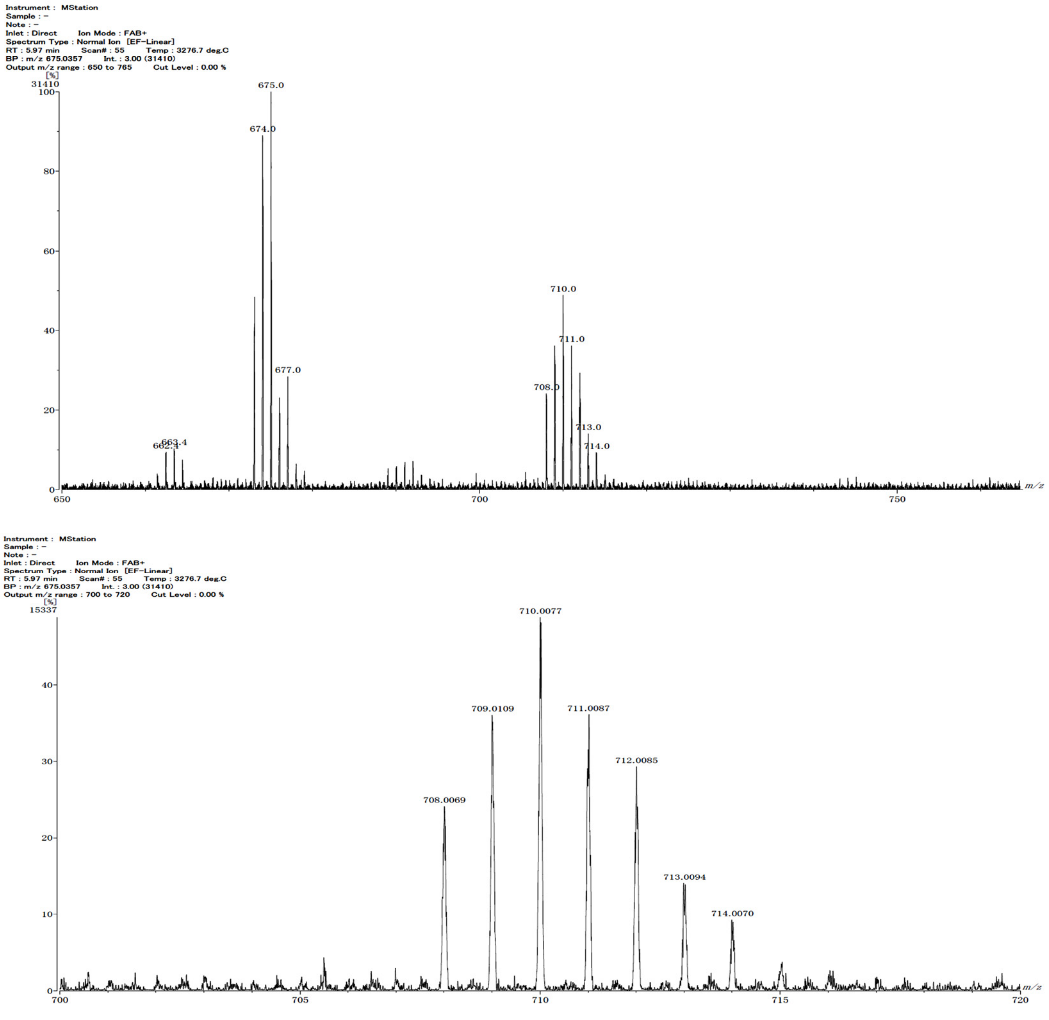

Figure S10. HR-FAB Mass of the compound TFBPC.

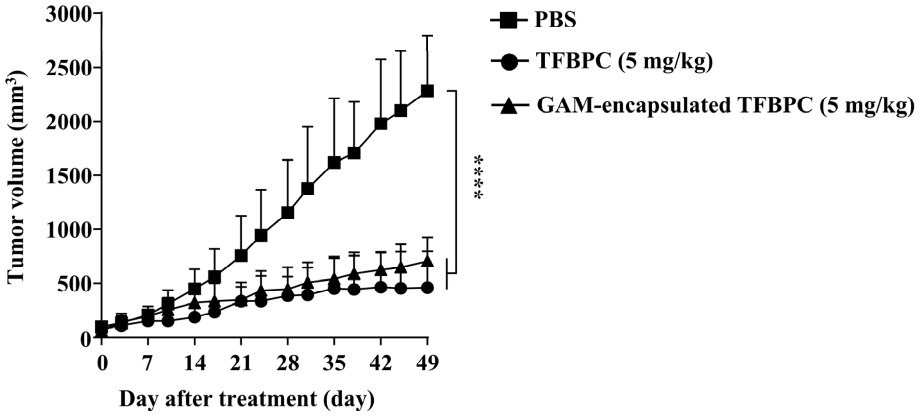

Figure S11. TFBPC and glycyrrhizic acid micelles (GAM)-encapsulated TFBPC significantly suppressed tumor growth in TNBC-bearing xenograft model. NSG mice (N = 16) with implantation of MDA-MB-231 cells on the left flank were intraperitoneally administrated with PBS (5 mL/kg), TFBPC (5 mg/kg) or glycyrrhizic acid micelles (GAM)-encapsulated TFBPC (5 mg/kg) once per week and four times total in the whole course of treatment. Tumor volume of each animal was measured by caliper for monitoring its tumor growth and then sacrificed 49 days after the first dose of treatment. Mean ± SD. **** $p < 0.0001$.

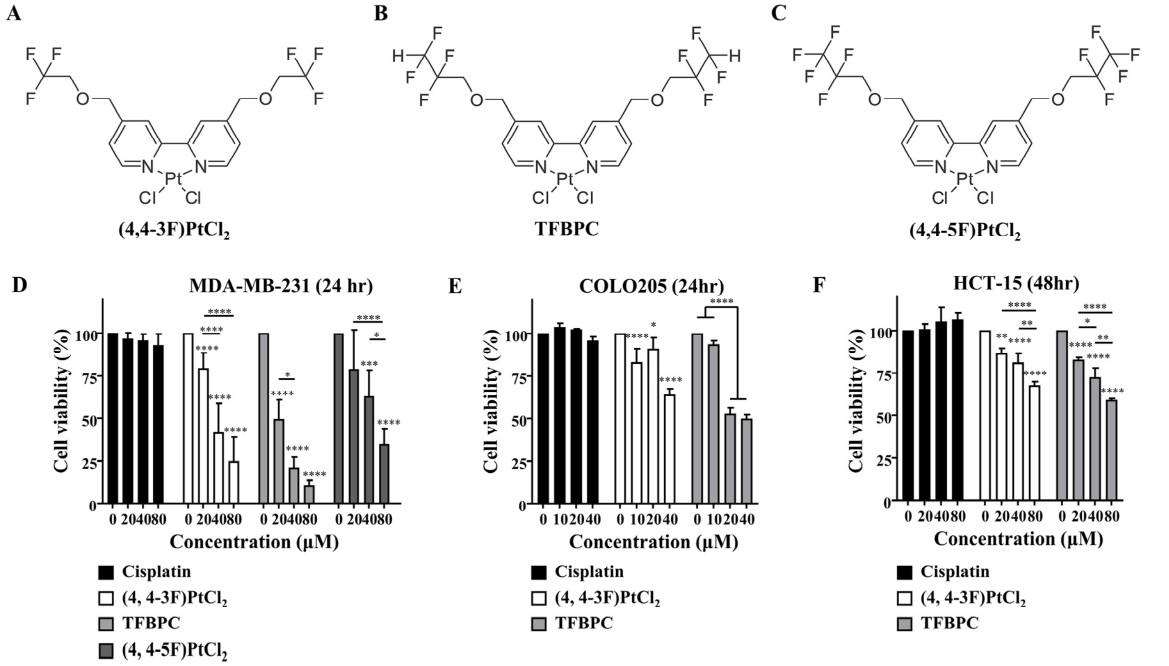

Figure S12. TFBPC significantly reduced cell survival rates in breast and colorectal cancers. Cisplatin and three platinum-based analogues, including (4, 4-3F) $PtCl_2$ (A), TFBPC (B) and (4, 4-5F) $PtCl_2$ (C), were assessed for cell viability using MTT assay. MDA-MB-231 breast cancers cells (D), COLO205 (E) and HCT-15 (F) colorectal cancer cells were given various concentrations of cisplatin or analogues ranging from 20 to 80 μM for 24 or 48 h. Results were shown in mean ± SD and statistically analyzed by one-way ANOVA and Tukey's post hoc test. * $p < 0.05$, ** $p < 0.01$, *** $p < 0.001$, **** $p < 0.0001$.

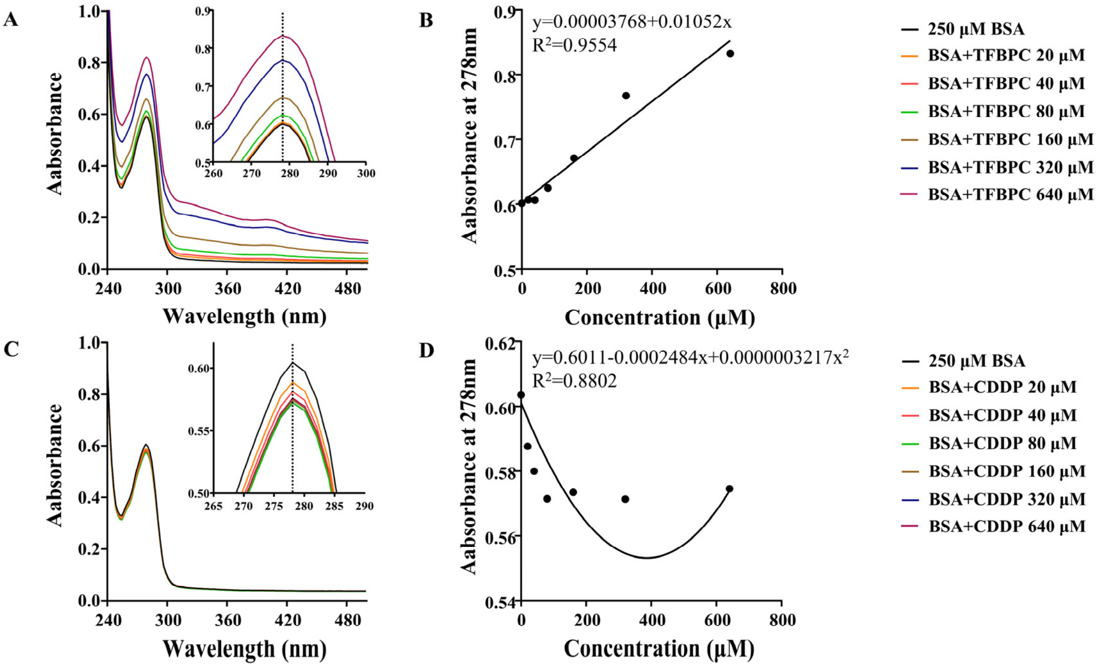

Figure S13. TFBPC interacted with bovine serum albumin and shifted the UV-visible spectra. Bovine serum albumin (BSA) in constant concentration at 250 μM was prepared in phosphate-buffered saline (PBS). The protein–drug interaction was explored by mixing different concentrations (20 to 640 μM) of platinum-containing compounds into BSA solution. The absorbance of BSA–drug mixtures which contain TFBPC (A) or cisplatin (C) was measured for UV-visible spectra (200 to 500 nm) via Thermo Varioskan Flash microplate reader. The vertical dotted line in the absorbance–wavelength plot demonstrated the maximal absorbance for pure BSA solution at 278 nm. The recorded absorbance at 278 nm for TFBPC (B) and CDDP (D) was plotted, and the equation of linear regression line or second-order polynomial (quadratic) trendline in TFBPC-BSA or CDDP-BSA interaction groups were shown.
